# The proton-sensing G protein-coupled receptor T-cell death-associated gene 8 (TDAG8) shows cardioprotective effects against myocardial infarction

**DOI:** 10.1038/s41598-017-07573-2

**Published:** 2017-08-10

**Authors:** Akiomi Nagasaka, Chihiro Mogi, Hiroki Ono, Toshihide Nishi, Yuma Horii, Yuki Ohba, Koichi Sato, Michio Nakaya, Fumikazu Okajima, Hitoshi Kurose

**Affiliations:** 1 Department of Pharmacology and Toxicology, Graduate School of Pharmaceutical Sciences, Kyushu University, Fukuoka, 812-8582 Japan; 2 Laboratory of Signal Transduction, Institute for Molecular and Cellular Regulation, Gunma University, 3-39-15 Showa-machi, Maebashi, 371-8512 Japan; 3 Laboratory of Signal Transduction, Faculty of Pharmaceutical Sciences, Aomori University, Aomori, 030-0943 Japan

## Abstract

Myocardial infarction (MI) is an ischaemic heart condition caused by the occlusion of coronary arteries. Following MI, lactic acid from anaerobic glycolysis increases and infiltrating immune cells produce severe inflammation, which leads to acidosis in the ischaemic heart. However, the physiological implication of this pH reduction remains largely unknown. T-cell death-associated gene 8 (TDAG8) is a proton-sensing G protein-coupled receptor found on cardiac macrophages that recognise increases in extracellular protons. We demonstrated that TDAG8 negatively regulates the transcription of the chemokine *Ccl20*. The infarcted hearts of TDAG8 KO mice showed an increase in CCL20 expression and the number of infiltrating IL-17A-producing γδT cells that express CCR6, a receptor for CCL20. Accordingly, excessive IL-17A production, which is linked to the functional deterioration after MI, was observed in MI-operated TDAG8 KO mice. The survival rate and cardiac function significantly decreased in TDAG8 KO mice compared with those in wild-type mice after MI. Thus, our results suggest that TDAG8 is a key regulator of MI and a potential therapeutic target.

## Introduction

Myocardial infarction (MI) is an ischaemic heart condition characterised by the occlusion of coronary arteries. Following MI, anaerobic respiration and severe inflammation occurs^1^. It is well known that anaerobic and inflammatory responses accompany an acidic environment, which is because of increased lactate and CO_2_ from anaerobic respiration and the pentose phosphate pathway, respectively^2, 3^. Previous studies showed that the pH in hearts of MI pigs, dogs and rabbits was reduced to 5.5–6.5^4–7^. However, the physiological importance of this pH reduction remains largely unknown.

Recent studies have reported that proton-sensing G protein-coupled receptors (GPCRs), which belong to the ovarian cancer G protein-coupled receptor 1 (OGR1) family, can recognise increases in extracellular protons and are completely activated at pH 6.4–6.8^8–11^. In 2003, Ludwig *et al*. first identified OGR1 and G protein-coupled receptor 4 (GPR4) as proton-sensing GPCRs. Subsequently, T-cell death-associated gene 8 (TDAG8, also known as GPR65) and G2 accumulation (G2A) were identified as proton-sensing GPCRs^10–12^. OGR1 has been implicated in asthma, prostate cancer and ovarian cancer, GPR4 has been implicated in malignant melanoma and angiogenesis, and G2A is involved in autoimmune diseases and leukaemia^13–15^. TDAG8 plays a role in inflammation by regulating the production of cytokines, including IL-6, TNF-α and IL-1β^16–19^, and in tumorigenesis by regulating cell survival and growth^20^.

The infiltration of immune cells into the infarct area has been implicated in the pathogenesis of several cardiovascular diseases, such as ischaemia/reperfusion (I/R) injury and MI^1, 21, 22^. The chemokine CCL20 (also known as MIP-3α) plays an important role in the migration of immune cells to the inflammation locus^23^. Specifically, the binding of CCL20 to the CCR6 receptor is responsible for the chemoattraction of leukocytes and IL-17A-producing γδT cells to the site of inflammation and thus the aggravation of cardiac function^24^.

We studied the physiological significance of pH reduction in MI by focusing on the function of the proton-sensing receptor TDAG8 using TDAG8 KO mice. These mice showed a decline in survival and cardiac function after MI in comparison with WT mice, indicating that sensing pH changes regulated by TDAG8 are physiologically important. TDAG8 KO mice also showed an increase in *Ccl20* transcription and the recruitment of CCR6^+^ IL-17A-producing γδT cells to the infarct area when compared with WT mice. Furthermore, an *in vitro* experiment with cardiac macrophages revealed that *Ccl20* transcription was, in part, negatively regulated in a TDAG8-dependent manner. Thus, our results suggest that TDAG8 exerts cardioprotective effects against MI by suppressing CCL20 expression and migration of CCR6^+^ IL-17A-producing γδT cells.

## Results

### TDAG8 expression was significantly increased in the infarct area after MI

**Figure 1:**
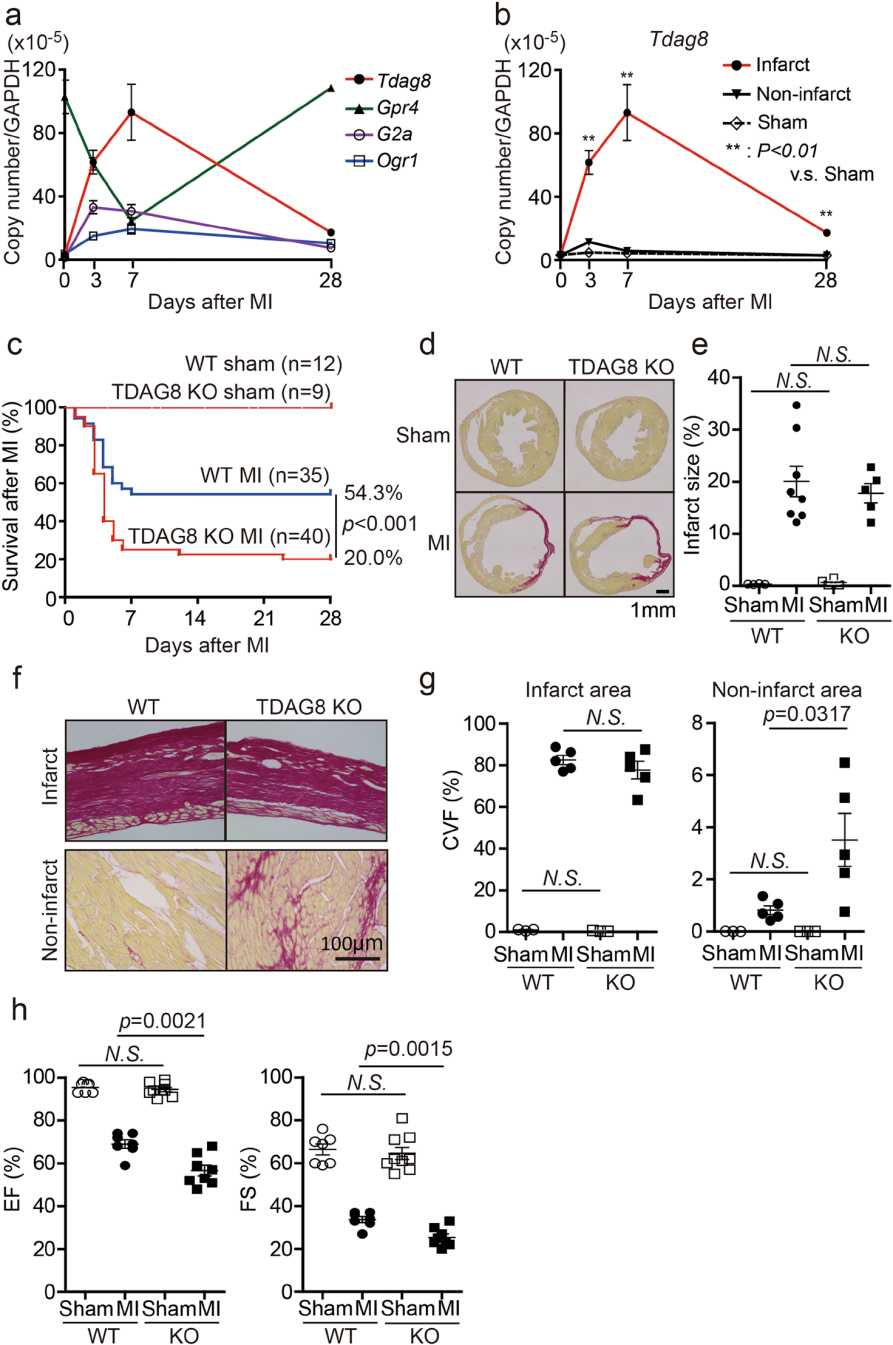
TDAG8 KO mice show high mortality in the early stages after MI. (**a**) Quantitative analysis of the expression of proton-sensing GPCR mRNAs in intact (n = 3) and infarcted WT hearts (n = 3–7). (**b**) Quantitative analysis of *Tdag8* mRNA levels in infarcted and non-infarcted areas of WT hearts after MI (sham n = 3; MI n = 3–7). Comparisons of group data were assessed with unpaired Student’s *t*-tests. Data are shown as the mean ± SEM. (**c**) Kaplan-Meier survival curve analysis in WT mice (sham n = 12, MI n = 35) and TDAG8 KO mice (sham n = 9, MI n = 40) after MI or a sham operation. The difference between WT-MI mice and TDAG8-KO mice was evaluated using the log-rank test. (**d**) Picrosirius Red staining of cardiac sections in MI- or sham-operated WT mice and TDAG8 KO mice after 28 days. (**e**) The infarct size was quantitatively estimated. (**f**) Representative heart sections of infarct areas and non-infarct areas 28 days after MI or sham procedures in WT mice (sham n = 3, MI n = 5) and TDAG8 KO mice (sham n = 3, MI n = 5) stained with Picrosirius Red. Positive staining indicates fibrosis. (**g**) The fibrotic area was quantified. (**h**) Echocardiography of WT mice (sham n = 7, MI n = 7) and TDAG8 KO mice (sham n = 9, MI n = 8) 28 days after MI or sham operation. Ejection fraction (EF) and fractional shortening (FS) results are shown. Comparisons were assessed using unpaired Student’s *t*-tests. Error bars represent the mean ± SEM (N.S., not significant).

Four types of proton-sensing GPCRs (TDAG8, OGR1, GPR4 and G2A) have been identified. However, the involvement of these receptors in the pathology of MI is largely unknown. Therefore, we examined the changes in the expression levels of these receptors after MI. MI-operated WT mice hearts were excised on the same day of the operation (day 0) and 3, 7 and 28 days after operation. We measured the mRNA levels of the four types of proton-sensing GPCRs in the heart samples using real-time RT-PCR. The mRNA expression of *Tdag8* most markedly increased after MI among the proton-sensitive receptors at the early phase of MI (Fig. 1a). In addition, the increase in *Tdag8* mRNA expression was observed in infarct areas but not in non-infarcted areas and sham-operated hearts (Fig. 1b). We also examined the effect of TDAG8 deficiency on the expression of proton-sensing GPCRs on post-MI day 3 between WT and TDAG8 KO mice. TDAG8 deficiency did not affect the expressions of other proton-sensing GPCRs (Supplementary Fig. S1). Based on these results, we assessed the role of TDAG8 in MI pathology.

### Declined survival and cardiac function in TDAG8 KO mice after MI

To examine the possible involvement of TDAG8 in the pathogenesis of myocardial infarction, we first compared the mortality rates between WT and TDAG8 KO mice on post-MI day 28. This comparison revealed that the survival rate on post-MI day 28 was 54.3% (19/35) in WT mice and 20.0% (8/40) in TDAG8 KO mice (Fig. 1c). Necropsy findings indicated that the main cause of death, which mainly occurred during post-MI days 3–7, was cardiac rupture. The infarct size (infarct area/total area) was similar between WT mice (20.05 ± 2.9%, n = 8) and TDAG8 KO mice (17.75 ± 1.9%, n = 5) on post-MI day 28 (Fig. 1d and e). To exclude the possibility that a difference in the degree of necrosis immediately after MI led to a difference in the incidence of cardiac rupture between groups, we investigated the initial infarct size 3 h after MI using Evans blue and 2,3,5-triphenyl tetrazolium chloride (TTC) staining of heart sections from WT mice and TDAG8 KO mice. The staining revealed that the size of the area at risk (AAR) and infarct area in WT mice and TDAG8 KO mice were similar (Supplementary Fig. S2). Subsequently, we determined the degree of fibrosis after MI, which has been identified as a critical factor in the development of heart failure^25, 26^. The degree of myocardial fibrosis in the infract area was similar between WT and TDAG8 KO mice; however, fibrosis in the non-infarct area was significantly exacerbated in TDAG8 KO mice compared with that in WT mice (Fig. 1f and g). Thus, the survivors on day 28 after operation were evaluated for their cardiac function by measuring the ejection fraction (EF) and fractional shortening (FS). The echocardiogram revealed that both EF and FS were significantly decreased in TDAG8 KO mice on post-MI day 28 compared with those in WT mice (Fig. 1h). These results suggested that TDAG8 deficiency leads to high mortality without affecting infarct size after MI and the TDAG8-mediated sensing of extracellular pH changes after MI is physiologically important for cardioprotection.

### Comparison of infiltrated immune cells in the infarcted hearts of WT and TDAG8 KO mice

**Figure 2:**
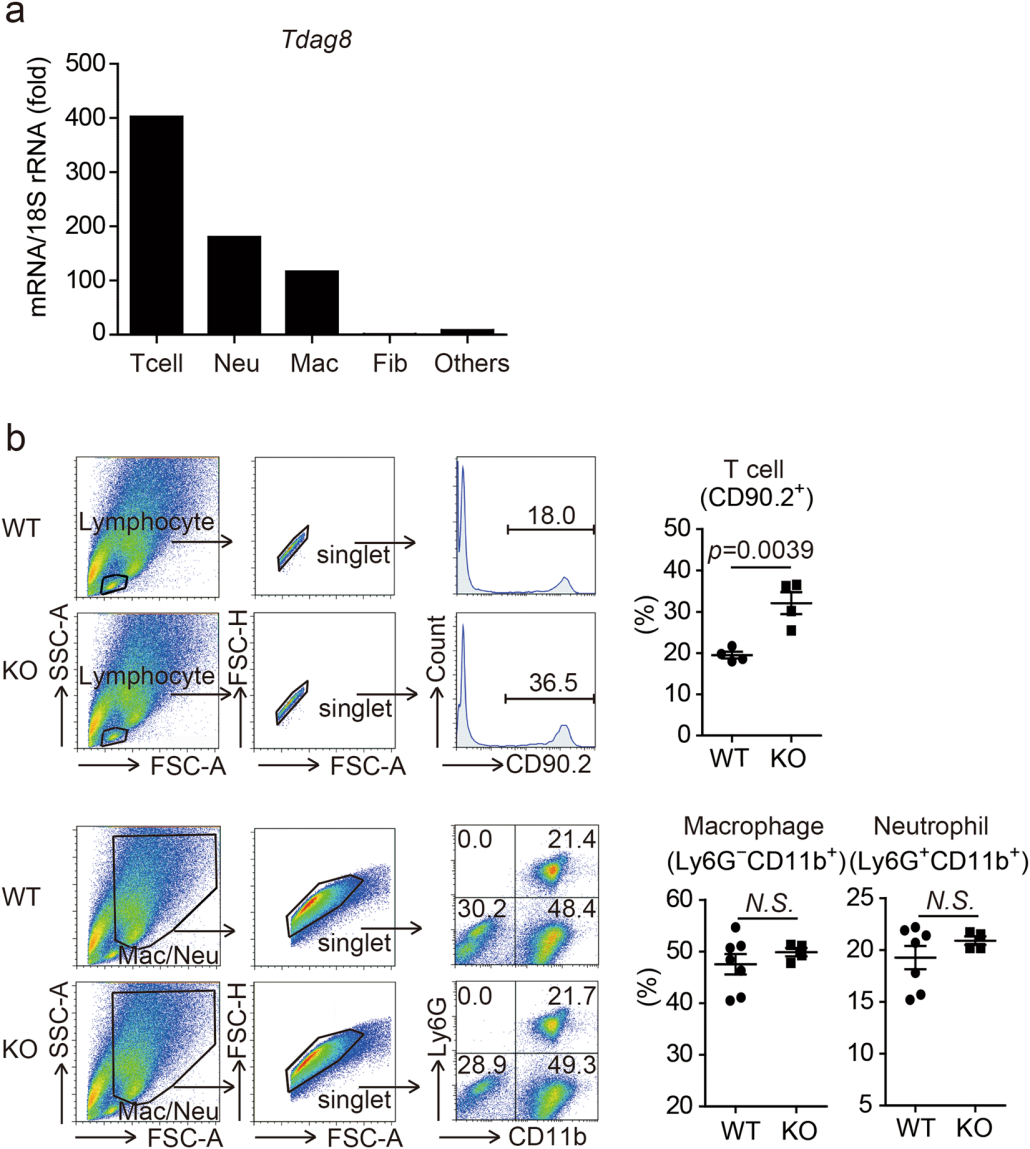
Comparison of TDAG8^+^ infiltrating cells in infarcted hearts of WT mice and TDAG8 KO mice. (**a**) *Tdag8* mRNA levels in T cells (T cell), neutrophils (Neu), macrophages (Mac), fibroblasts (Fib) and other cell types isolated from hearts of WT mice on post-MI day 3. Three infarcted WT mice hearts were stained with each antibody. After eliminating dead cells and gating singlet live cells, T cells (CD45.2^+^ Ly6G^−^ CD11b^−^ CD3^+^), neutrophils (CD45.2^+^ Ly6G^+^ CD11b^+^ CD3^−^), macrophages (CD45.2^+^ Ly6G^−^ CD11b^+^ CD3^−^), fibroblasts (CD45.2^−^ PDGFRα^+^) and other cells (CD45.2^−^ PDGFRα^−^) were sorted. The total mRNA collected from each cell population was used for real-time RT-PCR analysis. (**b**) Comparison of infiltrating T cells (CD90.2^+^), macrophages (Ly6G^−^ CD11b^+^) and neutrophils (Ly6G^+^ CD11b^+^) on post-MI day 3 between WT mice (n = 4–7) and TDAG8 KO mice (n = 4) Representative FACS profiles are shown. Comparisons were assessed using unpaired Student’s *t*-tests. Error bars represent the mean ± SEM (N.S., not significant).

We next identified TDAG8-expressing cells after MI. TDAG8-specific antibodies were not commercially available; therefore, we isolated cardiac cells on post-MI day 3 and measured TDAG8 expression using real-time RT-PCR. As previously reported^27^, TDAG8 was expressed in T cells, neutrophils and macrophages (Fig. 2a). In contrast, TDAG8 was expressed at low levels in fibroblasts and other fractions (e.g. myocytes). Based on our observation of TDAG8 expression levels in leukocytes, we compared the ratios of infiltrated immune cells in infarcted hearts on post-MI day 3 between WT and TDAG8 KO mice. The percentage of infiltrating T cells was significantly increased in TDAG8 KO mice compared with that in WT mice (Fig. 2b), whereas the percentage of infiltrating macrophages and neutrophils was similar between groups (Fig. 2b). Because the proportion of T cells was increased in TDAG8 KO mice, we examined whether the increase in T cells had an effect on the infiltration of B cells. Flow cytometry revealed that the percentage of B cells was decreased in TDAG8 KO mice (Supplementary Fig. S3). The percentage of B cells likely decreased because of the increased infiltration of T cells.

### Regulation of CCL20 expression by TDAG8

**Figure 3:**
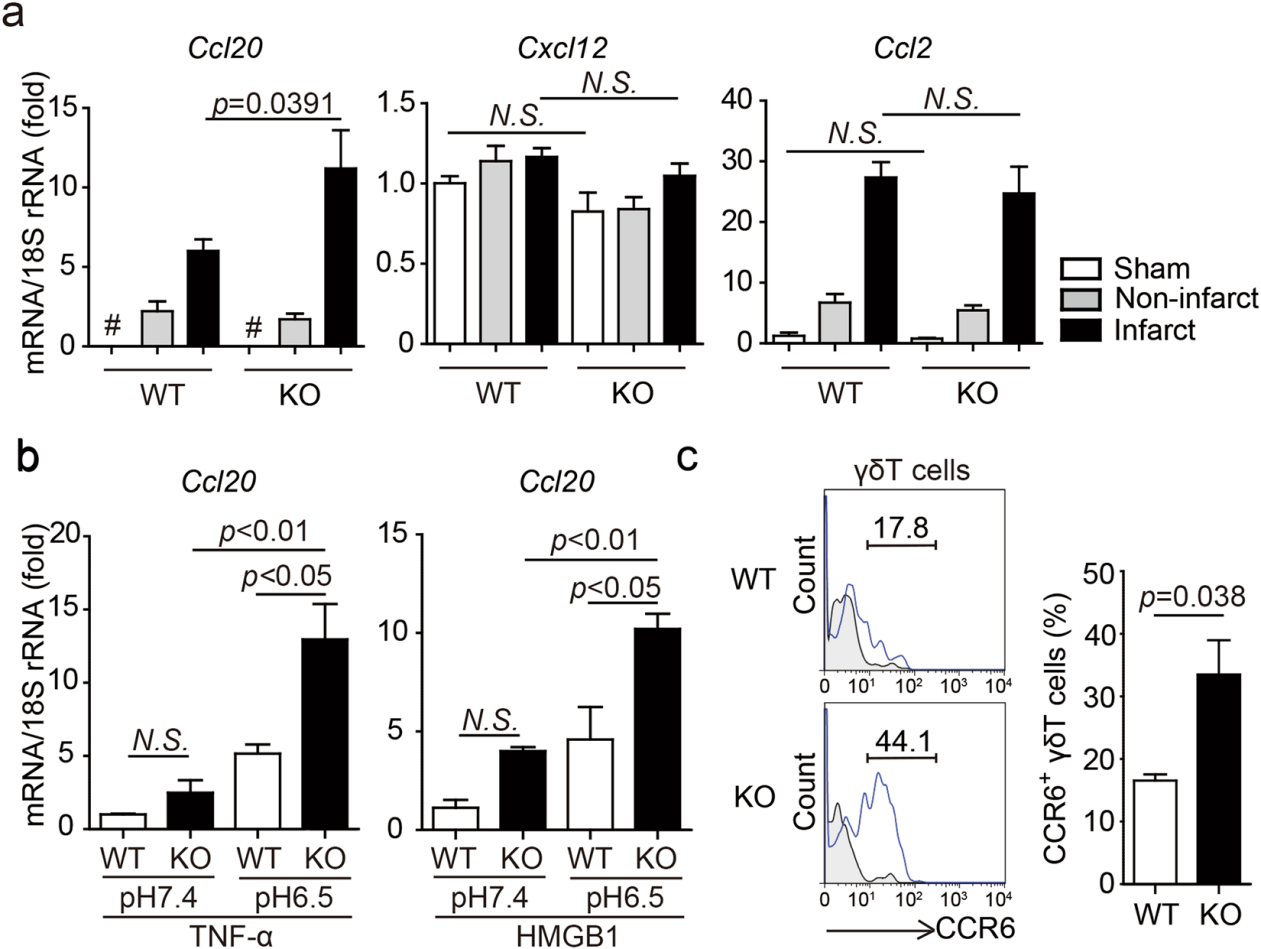
Partial regulation of *Ccl20* expression by TDAG8 on cardiac macrophages. (**a**) mRNA expression levels of chemokine genes in infarcted and non-infarcted areas of WT mice (sham n = 3, MI n = 7) and TDAG8 KO mice (sham n = 3, MI n = 5) 3 days after MI. ^#^ indicates that *Ccl20* mRNA was not detected. (**b**) *Ccl20* mRNA expression levels in cardiac macrophages treated with TNF-α or HMGB1 at pH 6.5 or pH 7.4. Cardiac macrophages were harvested from WT mice or TDAG8 KO mice 3 days after MI and treated with TNF-α (10 ng/ml) or HMGB1 (10 μg/ml) at pH 6.5 or pH 7.4 for 8 h to measure *Ccl20* mRNA using real-time RT-PCR. (**c**) Comparison of infiltrated CCR6^+^ γδT cells in infarcted hearts of WT and TDAG8 KO mice on post-MI day 3. The tinted histograms indicate the isotype control. Representative FACS profiles are shown. Comparisons were assessed using unpaired Student’s *t*-test (**a**,**c**) or a one-way ANOVA followed by Tukey’s test (**b**). Error bars represent the mean ± SEM (N.S., not significant).

The chemokines CCL20, CXCL12 and CCL2 are involved in the migration of T cells to inflammatory sites^24, 28, 29^. We compared the mRNA expression levels of these chemokines between WT and TDAG8 KO mice on post-MI day 3. As shown in Fig. 3a, the mRNA expression level of *Ccl20*, a chemokine that mediates γδT-cell recruitment^24^, was significantly higher in TDAG8 KO than in WT mice, whereas *Cxcl12* and *Ccl2* mRNA expression levels were similar between groups.

CCL20 is produced by endothelial cells and inflammatory cells, such as macrophages, stimulated with the inflammatory factors TNF-α and lipopolysaccharide (LPS)^23, 30^. As shown in Fig. 2b, the percentage of infiltrated macrophages was not different between WT and TDAG8 KO mice on post-MI day 3; however, *Ccl20* mRNA levels were significantly increased in TDAG8 KO mice. Moreover, no significant difference was found in the TNF-α expression levels between WT and TDAG8 KO mice (Supplementary Fig. S4). Based on these data, we hypothesised that TDAG8 on macrophages regulates CCL20. To test this hypothesis, first, we prepared macrophages from WT mice on post-MI day 3 to establish the time course of *Ccl20* mRNA expression in response to 10-ng/ml TNF-α or 10-μg/mL high-mobility group box 1 (HMGB1) under physiological conditions (pH = 7.4). HMGB1 binds toll-like receptor (TLR) 4 and TLR2 and play a crucial role in the initiation of inflammatory responses to I/R injury^31^. The maximal induction of *Ccl20* was observed 8 h after treatment (Supplementary Fig. S5). Next, we isolated cardiac macrophages from WT and TDAG8 KO mice on post-MI day 3 and treated them with TNF-α or HMGB1 for 8 h under acidic conditions (pH = 6.5) and physiological conditions (pH = 7.4). Consistent with our *in vivo* results, the *Ccl20* mRNA expression level under acidic conditions was markedly higher in TDAG8 KO mice than in WT mice (Fig. 3b). Unexpectedly, the *Ccl20* expression level was significantly higher in TDAG8 KO mice under acidic conditions than under physiological conditions. These results suggest that *Ccl20* transcription is regulated by molecules other than TDAG8, such as other proton-sensing GPCR(s).

The CCL20–CCR6 signalling axis is involved with the recruitment of γδT cells from lymph nodes after MI^24, 29^. Therefore, we investigated the percentage of CCR6^+^ γδT cells on post-MI day 3 and found that the infiltration of CCR6^+^ γδT cells was significantly increased in TDAG8 KO mice (33.5 ± 5.5%, n = 3) compared with that in WT mice (16.6 ± 1.0%, n = 3) (Fig. 3c). Similar results were obtained on post-MI day 5 (Supplementary Fig. S6).

### Enhanced recruitment of IL-17A-producing γδT cells and left ventricle remodelling in TDAG8 KO mice after MI

**Figure 4:**
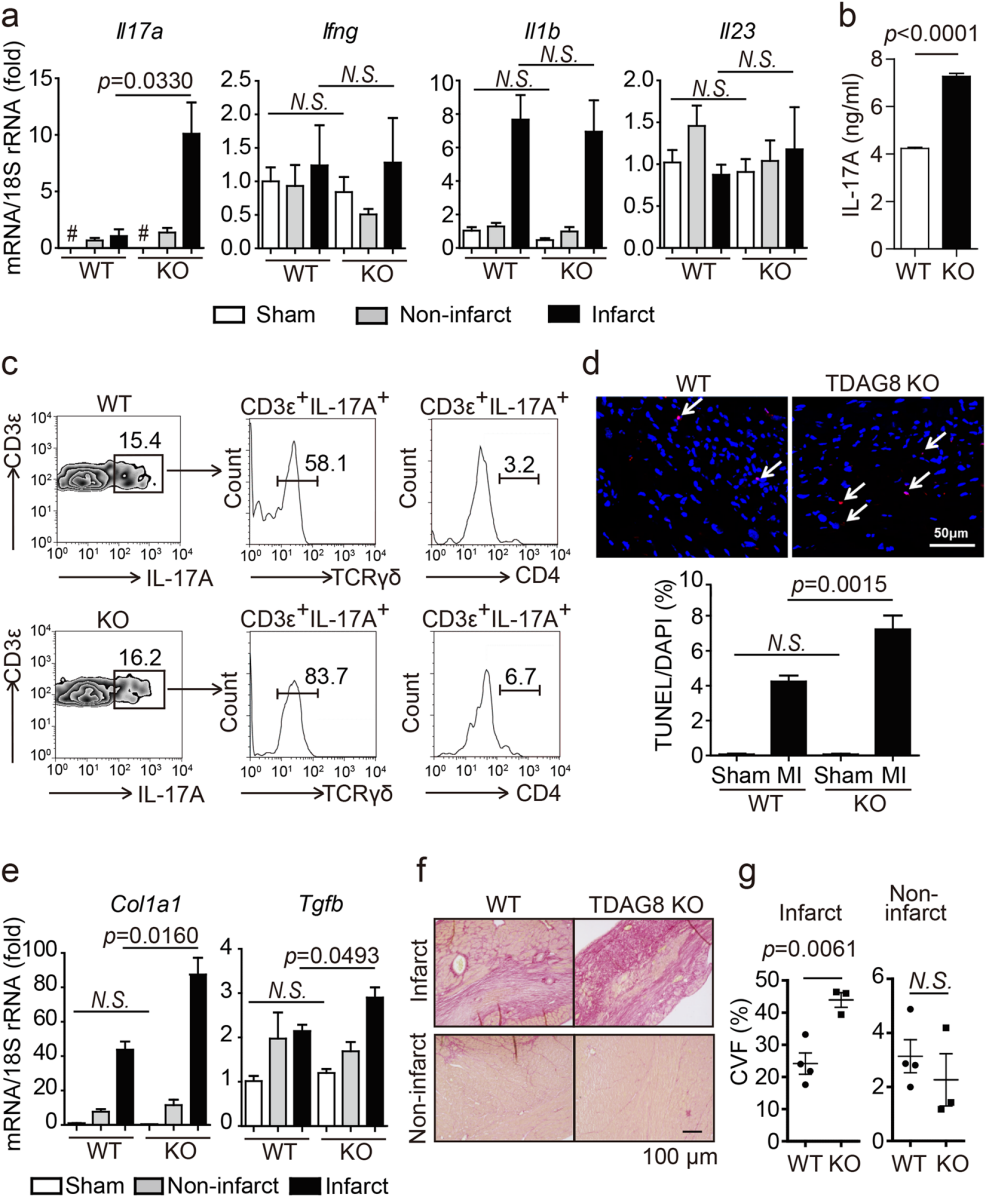
Enhancement of IL-17A-producing γδT cells and left ventricle remodelling in TDAG8 KO mice after MI. (**a**) mRNA expression levels of the indicated genes in infarcted and non-infarcted areas of WT mice (sham n = 3, MI n = 3–7) and TDAG8 KO mice (sham n = 3, MI n = 3–5) 3 days after MI or sham operation. # indicates that *IL-17a* mRNA was not detected. (**b**) All cardiac γδT cells isolated from WT and TDAG8 KO mice were treated with IL-1β (10 ng/ml) and IL-23 (10 ng/ml) on an anti-CD3 antibody-coated plate. Four infarcted hearts were combined for this analysis. After 48 h, the amount of secreted IL-17A in the supernatants was measured using ELISA. ELISA assay was performed in triplicates. (**c**) Infiltrated IL-17A-producing T cells in the infarcted hearts of WT mice and TDAG8 KO mice 3 days after MI were further analysed for γδTCR and CD4 expression using flow cytometry. Five heart samples were combined as one sample and used for analysis. (**d**) TUNEL staining of the infarcted area 3 days after MI. Tissue sections from WT mice (sham n = 3, MI n = 3) and TDAG8 KO mice (sham n = 3, MI n = 3) 3 days after MI were stained with TUNEL (red) and DAPI (blue). Arrows indicate apoptotic cells. (**e**) mRNA expression levels of fibrosis-related genes in infarcted and non-infarcted areas of WT mice (n = 3) and TDAG8 KO mice (n = 3) 7 days after MI or sham operation. Total mRNA extracted from sham-operated ventricles or MI-operated ventricles (separated into infarcted and non-infarcted areas) were subjected to real-time RT-PCR. (**f**) Representative heart sections of infarct and non-infarct areas 7 days after MI in WT mice (n = 4) and TDAG8 KO mice (n = 3) stained with Picrosirius Red. (**g**) The fibrotic area was quantified. Comparisons were assessed with unpaired Student’s *t*-tests. Error bars represent the mean ± SEM (N.S., not significant).

γδT cells secrete IL-17A and IFN-γ, both of which are involved in post-MI remodelling^24, 29^. Real-time RT-PCR results revealed that *IL-17a* mRNA expression level, but not *IFN-*γ mRNA expression level, was remarkably increased in TDAG8 KO mice on post-MI day 3 (Fig. 4a). We confirmed that *IL-17a* mRNA expression levels on post-MI day 7 remained significantly elevated in TDAG8 KO mice compared with that in WT mice (Supplementary Fig. S7). Although IL-1β and IL-23 are reported to play important roles in IL-17A production^32, 33^, no significant differences were detected in the expression levels of these two cytokines (Fig. 4a). In addition, we examined the mRNA expression levels of other cytokines mediated by IL-17A, including, IL-6, GM-CSF (also known as CSF-2) and IL-8 (also known as CXCL15)^34^. However, no significant differences in the expression of these cytokines were observed (Supplementary Fig. S8). Previous studies showed that the expression levels of IL-6 and GM-CSF did not change in IL-17A KO mice compared with in WT mice in an MI model and myocarditis model, respectively^24, 35^. Given these reports on the relationship between IL-17A and these inflammatory cytokines, our data suggests that an increased IL-17A expression is not necessarily involved in the production of these cytokines.

To confirm whether the difference in the number of infiltrating CCR6^+^ γδT cells lead to the difference in IL-17A production, we isolated cardiac γδT cells from WT and TDAG8 KO mice on post-MI day 3 and culture them in the presence of IL-1β and IL-23. After 48 h, IL-17A production was markedly higher in γδT cells from TDAG8 KO mice than from WT mice (Fig. 4b). Moreover, we examined the contribution of migrating γδT cells to IL-17A production and found that >25% of the migrating CCR6^+^ γδT cells in TDAG8 KO mice produced IL-17A (Supplementary Fig. S9). In addition to γδT cells, CD4^+^ T cells (Th17) can produce IL-17A, and CCR6 is involved in the migration of Th17 cells to inflammation sites^36^. To confirm the possible contribution of IL-17A from Th17 cells, we isolated cardiac cells on post-MI day 3 and stimulated them with PMA and ionomycin in the presence of Brefeldin A. More than 50% and 80% of IL-17A-producing T cells were CD4^−^ γδT cells in WT and TDAG8 KO mice, respectively (Fig. 4c). Th17 cells comprised <10% of IL-17A-producing cells, indicating that γδT cells, rather than Th17 cells, are the major source of IL-17A. Accordingly, these results suggest that increased *IL-17a* expression in TDAG8 KO mice after MI is partly because of the enhanced infiltration of IL-17A-producing CCR6^+^ γδT cells via CCL20.

Excessive IL-17A expression leads to cardiomyocyte apoptosis and fibrosis after MI^24, 33^. Thus, we completed terminal deoxynucleotidyl transferase-mediated dUTP nick end-labelling (TUNEL) on post-MI day 3 to detect apoptotic cells. As shown in Fig. 4d, TDAG8 KO mice exhibited a marked increase in the number of TUNEL-positive cells (7.2 ± 0.8%, n = 3) compared with that in WT mice (4.2 ± 0.4%, n = 3). Next, we examined the degree of fibrosis between WT and TDAG8 KO mice on post-MI day 3, the initial phase of fibrosis. Real-time RT-PCR analysis and Picrosirius Red staining demonstrated that the expression levels of fibrosis-related genes, such as *Col1a1* and *Tgfb1*, and the degree of collagen content in the infarct areas were comparable between groups (Supplementary Fig. S10). In contrast, on post-MI day 7, i.e., when fibrosis is highly accelerated, the expression levels of fibrosis-related genes were significantly higher in TDAG8 KO mice than in WT mice (Fig. 4e). Consistent with the real-time RT-PCR results, fibrosis in the infarct area was substantially enhanced in TDAG8 KO mice (49.3 ± 2.3%, n = 3) compared with that in WT mice (24.2 ± 3.3%, n = 4); however, the extent of fibrosis was similar in the non-infarct areas between WT and TDAG8 KO mice (Fig. 4f and g). No WT mice exhibited fibrosis with a severity that was observed in TDAG8 KO mice. Thus, the observed enhancement of fibrosis is unbiased because of the low survival rate of TDAG8 KO mice. The TDAG8 KO mice surviving 7 days after MI are most likely to exhibit accelerated fibrosis and a subsequent decrease in cardiac function on post-MI day 28 (Fig. 1f–h). Taken together, these results suggest that the enhanced production of IL-17A by γδT cells is associated with the increased apoptosis and fibrosis. These may lead to the worsening of survival rate, cardiac function and remodelling after MI in TDAG8 KO mice.

**Figure 5:**
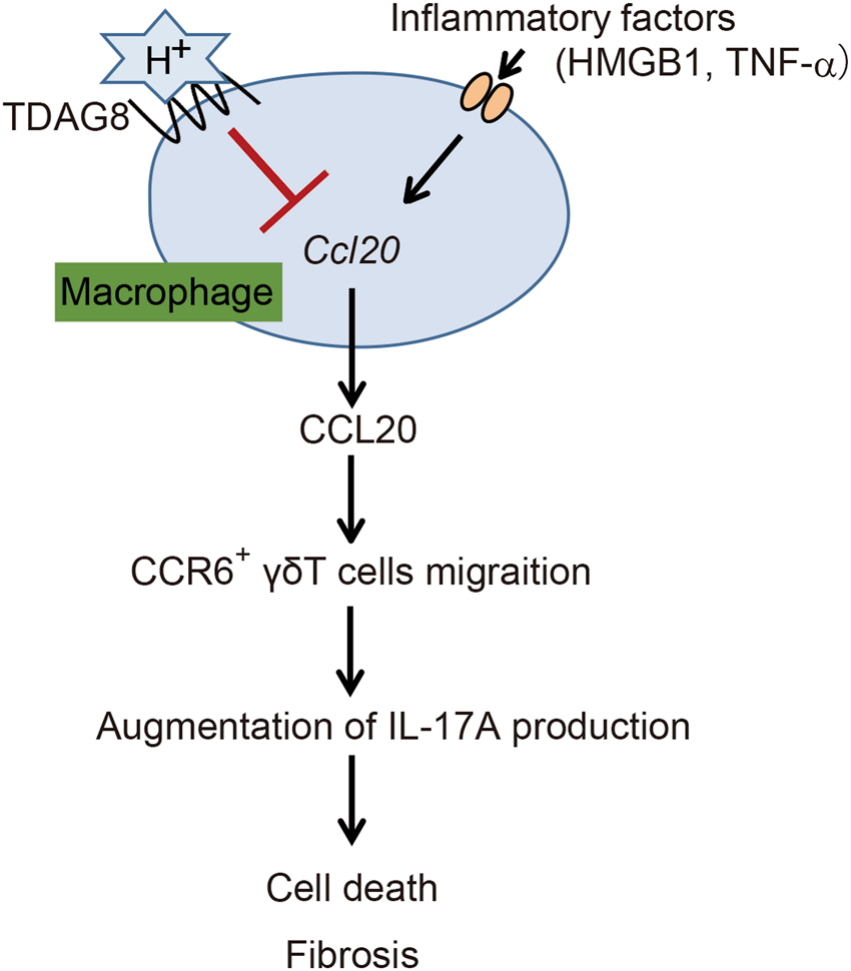
Postulated model showing the cardioprotective effects of TDAG8-expressing cardiac macrophages against MI. MI induces a pH reduction and severe inflammation at infarcted areas. TDAG8 expressed on cardiac macrophages recognises extracellular pH changes and inhibits the *Ccl20* transcription stimulated by inflammatory factors (e.g. HMGB1 and TNF-α), which inhibits the recruitment of IL-17A-producing CCR6^+^ γδT cells. These cells are mediators of cell death and fibrosis, thus explaining the cardioprotective effects of TDAG8.

Thus, TDAG8 found on macrophages detects acidic microenvironments and suppresses *Ccl20* transcription in MI, which may lead to the inhibition of the recruitment of IL-17A-producing CCR6^+^ γδT cells to the infarct area. As these cells mediate fibrosis and apoptosis, in part, their inhibition could result in the cardioprotective effects of TDAG8 (Fig. 5).

## Discussion

Extracellular acidosis is frequently associated with several ischaemic and inflammatory diseases, such as ischaemic heart disease, stroke, asthma, rheumatoid arthritis and solid tumours^9^. In ischaemic heart disease, lactic acid from anaerobic glycolysis increases and leukocytes induce severe inflammation of the infarcted area, which lead to extracellular acidosis and deterioration of cardiac function after MI^1, 9, 37^. In the present study, we determined that TDAG8 showed cardioprotective effects against in MI. This effect was characterised by increased *Ccl20* transcription in macrophages and recruitment of CCR6^+^ IL-17A-producing γδT cells at the infarct area. Recent studies have reported that γδT cell infiltration and IL-17A production occurs at the early stages of MI and peaks on post-MI day 7^24, 33^. The levels of IL-17A production, which is linked to cardiomyocyte apoptosis and fibrosis, is accompanied with γδT cell infiltration into the infarcted hearts. Consistent with these reports, we showed that γδT cells were the major source of IL-17A in TDAG8 KO and WT mice during this stage and that the IL-17A production was significantly enhanced in the infarcted hearts of TDAG8 KO mice, accompanied by increased numbers of γδT cells. The enhancement of cardiac apoptosis, which is a factor of induction of cardiac rupture^38, 39^, was observed in the infarcted hearts of TDAG8 KO mice on post-MI day 3 (Fig. 4d). However, we have not shown that overproducing IL-17A directly induces apoptosis in cardiomyocytes. It remains to be determined if the increase of cardiac rupture in TDAG8 KO mice after MI is caused by the enhancement of cardiomyocyte cell death by IL-17A. In addition, fibrosis during the early stage of MI is crucial for preventing cardiac rupture^26^. We showed that fibrosis was comparable between the WT and TDAG8 KO mice on post-MI day 3 (Supplementary Fig. 10). It is assumed that the promotion of fibrosis by IL-17A was not observed on post-MI day 3 because the number of collagen-producing cells (fibroblasts/myofibroblasts) was still small on that day^26, 40^. Collectively, these results suggested that the decline in the survival rate because of cardiac rupture in MI-operated TDAG8 KO mice (Fig. 1c) was partly due to the enhanced apoptosis compared with that in the WT mice despite the same level of collagen accumulation.

We determined that cardiac macrophages isolated from TDAG8 KO mice significantly increased *Ccl20* transcription under acidic conditions when compared with physiological conditions. This result suggests that molecules, such as other proton-sensing GPCR(s), accelerate *Ccl20* transcription under acidic conditions. Indeed, we determined that G2A was highly expressed on cardiac macrophages (Supplementary Fig. S11). Although G2A recognises pH fluctuations and mediates inositol phosphate accumulations in a pH-dependent manner^11^, the ligand for G2A remains controversial^9^. Hattori *et al*. reported that oxidised free fatty acids were another type of G2A ligands and that 9-hydroxyoctadecadienoic acid, an oxidised fatty acid, stimulated the secretion of inflammatory cytokines, such as IL-6, IL-8 and GM-CSF, through G2A in normal human keratinocytes^41^. Thus, it would be intriguing to examine whether G2A mediates pH-dependent *Ccl20* expression in cardiac macrophages.

TDAG8 transduces signals via the Gs/cAMP/PKA or G_12/13_/Rho signalling pathways^10, 12, 27^. Recently, Jin *et al*. reported that the transcriptional regulation of LPS-induced IL-1β in mouse microglia was suppressed through the TDAG8/Gs/cAMP/PKA pathway by inhibiting extracellular signal-related kinase (ERK), c-Jun *N*-terminal kinase and activator protein (AP)-1 binding^19^. The *Ccl20* promoter region in humans contains several transcription binding sites, including but not limited to nuclear factor (NF)-κB, AP-1, CCAAT/enhancer-binding proteins (C/EBP) and specificity protein 1 (Sp1)^42^. Several studies demonstrated that NF-κB, C/EBPβ and Sp1 are transcriptional regulators of CCL20 in various cell types^42–45^. Thus, these transcriptional factors could be involved in the TDAG8-dependent transcriptional regulation of CCL20 expression in cardiac macrophages.

We demonstrated that TDAG8 exerted cardioprotective effects against MI, highlighting its potential role as a therapeutic target. TDAG8 agonists have been reported^20, 46^. Therefore, TDAG8 agonists may be useful to treat diseases associated with pH reduction and recruitment of IL-17A-producing γδT cells, such as MI, stroke and rheumatoid arthritis^28, 47^. A recent study reported that higher serum levels of CCL20 were observed in patients with ischaemic heart disease when compared with healthy controls^48^. Furthermore, the administration of a CCL20-neutralising antibody improved the prognosis in CVB3-induced acute viral myocarditis by decreasing CCR6^+^ Th17 cell recruitment^49^. Taken together, these studies support the hypothesis that CCL20-neutralising antibodies administered to patients with ischaemic heart disease have the potential to suppress the migration of CCR6^+^ IL-17A-producing γδT cells and improve prognosis. Furthermore, a recent study has reported that tumour-associated macrophages (TAMs) can produce CCL20, resulting in increased recruitment of CCR6^+^ regulatory T cells, and promote colorectal cancer development in mice^50^. CCL20 is also expressed in pancreatic carcinoma, renal cancer, breast carcinoma, pancreatic cancer and papillary thyroid carcinoma^51^. In general, high numbers of TAMs are associated with poor prognosis^51, 52^. We demonstrated that TDAG8 on cardiac macrophages negatively regulates CCL20 expression; thus, this finding can be applied to understand the involvement of CCL20 production in TAMs. TDAG8 has the potential to be a therapeutic target in these cancers.

## Methods

### Mice and surgical procedures for MI

We purchased C57BL/6 J mice from SLC (Japan). TDAG8 KO mice (backcrossed for >8 generations on the C57BL/6 background) were obtained from Dr. Satoshi Ishii (Akita University, Japan) and Dr. Takao Shimizu (Tokyo University, Japan). Male mice (8–12 weeks old) were subjected to permanent left anterior descending coronary artery ligation under anaesthesia. The sham-operated animals underwent the same procedure without ligation of the coronary artery. All animal experiments were performed in accordance with the relevant national and international guidelines contained in the ‘Act on Welfare and Management of Animals’ (Ministry of Environment of Japan) and ‘Regulation of Laboratory Animals’ (Kyushu University), and their protocols were approved by the Institutional Animal Care and Use Committee at Kyushu University.

### Echocardiography

Echocardiographic and haemodynamic parameters were measured as previously described^21^. We performed transthoracic ultrasound cardiography on post-MI day 28 day under anaesthesia. Two-dimensional targeted M-mode tracings were recorded using a Nemio GX image analysing system (SSA-580A, Toshiba Medical Systems). The percent EF (% EF) was calculated using Pombo’s method. To estimate cardiac systolic function, the percent fractional shortening (% FS) was calculated: % FS = [(LVIDd − LVIDs)/LVIDd] × 100.

### Analysis of fibrosis

Excised hearts were fixed with 4% paraformaldehyde (PFA) in 0.1-M phosphate buffer containing 4% sucrose at 4 °C for >12 h. After a gradual dehydration with ethanol and xylene, hearts were embedded in paraffin and sectioned at 5 μm using a microtome (RM2155; Leica). The paraffin sections were deparaffinised by dipping into xylene and ethanol and then stained with Picrosirius Red for 1 h. After washing with 0.01-N HCl, sections were dehydrated with ethanol and xylene and mounted with Mount-Quick (Daido Sangyo). Sections were analysed using fluorescence microscopy (BioRevo BZ-9000; KEYENCE). The collagen volume fraction was calculated using a BZ-II analyser (KEYENCE). For post-MI day 3 samples, frozen sections of 6 µm thickness were prepared. The same method described above was used for Picrosirius Red staining.

### Infarct size assessment

The infarct size was assessed as previously reported^53^. Briefly, 3 h after MI, anesthetised mice were injected in the abdominal aorta with 1 ml of 2% Evans blue (Wako). Next, the heart was excised, and sliced were obtained and soaked in 1% TTC (Sigma) for 15 min at 37 °C and then immersed into 10% formalin neutral buffer solution (Wako) to preserve overnight at 4 °C. The infarct area (TTC-negative) was represented by the ratio of the AAR (area not stained with Evans blue). The AAR was represented as a ratio of the left ventricle (LV) area.

### Isolation of infiltrated cells from infarcted hearts

To confirm the types of immune cells that infiltrated infarcted hearts, mice hearts were excised 3 days after the operation. After removing the atria, the ventricles were digested in PBS containing 0.2% collagenase type II (Worthington) and 0.02% elastase (Worthington) at 37 °C for 10 min with agitation. The digestion process was repeated 3 times. For the assessment of CCR6 expression in γδT cells and the isolation of cardiac macrophages for the *in vitro* experiment, samples were digested in PBS containing 0.1% collagenase A (Roche), 0.1% trypsin (Sigma) and 0.1% BSA (Sigma) at 37 °C for 10 min with agitation. The digestion process was repeated 10 times. Isolated cells were treated with Red Blood Lysis buffer (Roche).

### Flow cytometry

After isolated cardiac cells were treated with mouse Fc Block^TM^ (BD), samples were stained with anti-CD45.2-Alexa Fluor 488 (BioLegend), anti-CD45.2-APC (BioLegend), anti-Ly6G-PE-Cy7 (BioLegend), anti-CD11b-PerCP-Cy5.5 (BioLegend), anti-CD11b-PE (BD), anti-PDGFRα-APC (BioLegend), anti-CD3ε-PE (BD), anti-CD3ε-PE-Cy5 (TONBO Biosciences), anti-CD4-APC-Cy7 (TONBO Biosciences), anti-CD90.2-APC (eBiosceince), anti-γδTCR-PE (BD), anti-CCR6-APC (RD Systems), anti-B220-APC (BioLegend). For IL-17A staining, cell surface staining was performed, followed by fixation and permeabilisation with Cyofix/Cytoperm^TM^ (BD) and staining for anti-IL-17A-PE-Cy7 (eBioscience). Dead cells were labelled with the eFluor 780 viability dye (eBioscience). Stained samples were analysed using flow cytometry (AriaIII; BD).

### Real-time RT-PCR

Total RNA was extracted from sham- or MI-operated hearts using ISOGEN (Nippon Gene) and a RNeasy Plus Mini Kit (Qiagen) according to the manufacturer’s instructions. Total RNA was extracted from cardiac macrophages and sorted cells using ISOGEN (Nippon Gene) and the Gene-Packman Coprecipitant (Nacalai Tesque) according to the manufacturer’s instruction. cDNA was synthesised using a High Capacity cDNA Reverse Transcription Kit (Thermo Fisher). mRNA expression was quantified using a StepOnePlus^TM^ real-time PCR System (Thermo Fisher). Data were normalised to 18 S rRNA or GAPDH. Specific primers and probes used in this study are listed in Supplementary Table 1.

### TUNEL staining

Excised hearts were fixed with 4% PFA in 0.1-M phosphate buffer containing 4% sucrose at 4 °C for 2 h. The fixed hearts were incubated in PBS containing 10% and 20% sucrose for 4 h or overnight, respectively. Next, the samples were embedded in OCT compound (Sakura Finetek). Frozen specimens were sliced at 6 μm using a cryostat. Cryostat sections were analysed with a ApopTag^TM^ Red *In Situ* Apoptosis Detection Kit (Millipore) according to the manufacturer’s instructions. Sections were mounted with 2-μg/ml 4′,6-diamino-2-phenylindole (DAPI) containing FluorSave^TM^ reagent (Milipore). Five randomly selected fields from left ventricles in sham-operated mice and infarcted areas in MI-operated mice were assessed with a confocal microscope (LSM700; Carl ZEISS). The number of TUNEL-positive nuclei was normalised to the total DAPI-positive nuclei in the same section using ZEN Imaging Software (Carl ZEISS).

### *In vitro* cardiac cell stimulation

For *in vitro* cardiac γδT-cell stimulation, prepared cardiac cells from infarcted hearts were treated with 50-ng/ml phorbol 12-myristate 13-acetate (PMA) and 1-μg/ml ionomycin in the presence of 5-μg/ml Brefeldin A in RPMI-1640 supplemented with 10% FBS and 2-mM glutamine at 37 °C for 4 h. The samples were analysed with flow cytometry. For *in vitro* cardiac macrophage stimulation, cells were seeded at a density of 2 × 10^5^ cells/well in DMEM supplemented with 10% FBS and antibiotics in 24-well plates at 37 °C for 4 h. Four hours after incubation, the culture medium was changed to a low-serum media containing 0.5% FBS to remove fibroblasts and non-adherent cells. Cells were incubated at 37 °C for 18 h. Next, the cardiac macrophages were treated with 10-ng/ml TNF-α (PeproTech) or 10-μg/ml bovine HMGB1 (Chondrex) at 37 °C for 8 h in DMEM containing 0.5% FBS, antibiotics and 25-mM HEPES at a pH = 6.5 or pH = 7.4. The pH of the medium was adjusted with HCl or NaOH.

### ELISA

Cardiac γδT cells were enriched from total cardiac cells using anti-PE Microbeads UltraPure (Miltenyi Biotec) after staining with PE-conjugated anti-mouse γδTCR (BD). Isolated cardiac γδT cells were transferred to 96-well Maxisorp plate (Nunc), which was coated overnight with 100 μl of 4-μg/ml anti-CD3 antibody (145-2C11; TONBO Biosciences) and cultured in the presence of 10-ng/ml mIL-1β (Peprotech) and 10-ng/ml mIL-23 (RD systems). After 48 h, the supernatants were collected and assayed for IL-17A production using LEGEND MAX^TM^ ELISA Kit (BioLegend) according to the manufacturer’s instructions.

### Statistical analysis

We used GraphPad Prism 6.0 software for all statistical analyses. The results are represented as the mean ± standard error of mean from at least three independent experiments. Statistical analyses were performed using an unpaired Student’s *t*-test for two-group comparisons and a one-way ANOVA followed by the Tukey or Newman-Keuls test for multiple group comparisons. The statistical significance was set at *P* < 0.05.

## Electronic supplementary material

supplementary information and figures
