# Supplementary material for: The proton-sensing G protein-coupled receptor T-cell death-associated gene 8 (TDAG8) shows cardioprotective effects against myocardial infarction
Source: Sci Rep. 2017 Aug 10;7:7812. doi: 10.1038/s41598-017-07573-2 (PMC5552703; doi:10.1038/s41598-017-07573-2)
Supplement: Supplementary file 1 — supplementary information and figures [file 41598_2017_7573_MOESM1_ESM.pdf]

Supplementary information

The proton-sensing G protein-coupled receptor T-cell death-associated gene 8 (TDAG8) shows cardioprotective effects against myocardial infarction

Akiomi Nagasaka[1+], Chihiro Mogi[2+], Hiroki Ono[1+], Toshihide Nishi[1], Yuma Horii[1], Yuki Ohba[1], Koichi Sato[2], Michio Nakaya[1], Fumikazu Okajima[2, ¶], Hitoshi Kurose[1,*]

Supplementary Figure 1. Comparison of Gpr4, Ogr1 and G2a expression levels in the hearts of WT and TDAG8 KO mice on post-MI day 3.

Quantitative analysis of the expression of proton-sensing GPCR mRNAs in WT mice hearts (sham, n = 3; MI, n = 7) and TDAG8 KO mice hearts (sham, n = 3; MI, n = 5) on post-MI day 3. mRNA expression was normalized to GAPDH. Comparisons were assessed using unpaired Student's *t*-tests. Error bars represent the mean ± SEM (N.S., not significant).

Supplementary Figure 2. The area at risk and infarct size were comparable between WT and TDAG8 KO mice during the initial phase after MI.

(a) Representative heart sections of Evans blue/TTC staining 3 h after MI. The non-ischemic area is indicated in blue, the area at risk (AAR) in red and the infarct area in white. (b) The infarct size and AAR were quantified as a percentage of the AAR and the left ventricular area (LV) of WT (n = 5) and TDAG8 KO (n = 4) mice. Error bars represent the mean ± SEM. Comparisons among groups were assessed using unpaired Student's *t*-test (N.S., not significant).

Supplementary Figure 3. Comparison of infiltrated B cells in the infarcted hearts

of WT and TDAG8 KO mice.

Comparison of infiltrated B cells (B220$^+$) on post-MI day 3 between WT mice (n = 4) and TDAG8 KO mice (n = 3) was performed using flow cytometry. Representative FACS profiles are shown. The comparison was assessed using unpaired Student’s *t*-tests. Error bars represent the mean ± SEM.

Supplementary Figure 4. No significant differences in *Tnfα* mRNA were found on post-MI day 3.

Tnfα mRNA expression in infarcted and non-infarcted areas of WT mice (sham n = 3, MI n = 7) and TDAG8 KO mice (sham n = 3, MI n = 5) 3 days after MI was measured using real-time RT-PCR. The comparison was assessed using an unpaired Student’s *t*-test.

Supplementary Figure 5. Time course of *Ccl20* mRNA expression in cardiac macrophages stimulated with TNF-α and HMGB1.

Cardiac macrophages were isolated from WT mice on post-MI day 3 and stimulated with 10-ng/ml TNF-α (left) or 10-μg/ml HMGB1 (right). Three heart samples were combined as one sample and used for real-time RT-PCR analysis.

Supplementary Figure 6. Comparison of infiltrated CCR6^{+} γδT cells in the infarcted hearts of WT and TDAG8 KO mice on post-MI day 5.

Cardiac γδT cells were isolated from WT and TDAG8 KO mice 5 days after MI; stained with antibodies against CD3ε, γδTCR and CCR6; and were analysed by flow cytometry. Grey histograms indicate the isotype control.

Supplementary Figure 7. Upregulation of *IL-17a* mRNA in TDAG8 KO mice 7 days after MI.

IL-17a mRNA expression in the non-infarct and infarcted areas of WT mice (n = 7) or TDAG8 KO mice (n = 5) 7 days after MI was measured using real-time RT-PCR. Comparisons were assessed with a one-way ANOVA followed by Tukey's test. Error bars represent the mean ± SEM (N.S., not significant).

Supplementary Figure 8. Comparison of IL-17A-related inflammatory genes of WT and TDAG8 KO mice.

Expression levels of inflammatory gene mRNAs in the infarcted and non-infarcted areas of WT mice (sham, n = 6; MI, n = 10) and TDAG8 KO mice (sham, n = 5; MI, n = 8)

on post-MI day 3. # indicates that *Il8* mRNA was not detected. Comparisons were assessed using unpaired Student’s *t*-tests. Error bars represent the mean ± SEM (N.S., not significant).

Supplementary Figure 9. A portion of CCR6^{+} γδT cells secrete IL-17A.

Cardiac cells were isolated from TDAG8 KO mice on post-MI day 5 and stimulated with PMA and ionomycin in the presence of Brefeldin A. The samples were stained with antibodies against CD3ε, γδTCR, CCR6 and IL-17A. After gating visible and singlet cells, CD3ε^{+} γδCR^{+} CCR6^{+} cells were further gated, and IL-17A expression was determined using flow cytometry. Mixed samples (n = 3) were used for the analysis.

Supplementary Figure 10. Comparison of fibrosis between WT and TDAG8 KO mice on the post-MI day 3.

(a) Expression levels of fibrosis-related gene mRNAs in the infarcted and non-infarcted area of WT mice (sham n = 3, MI n = 7) and TDAG8 KO mice (sham n = 3, MI n = 5) on post-MI day 3. (b) Quantification of the fibrotic area using Picrosirius Red staining in the infarcted areas on post-MI day 3 in WT (n = 3) and TDAG8 KO (n = 3) mice. Comparisons were assessed using unpaired Student’s *t*-tests. Error bars represent the

mean ± SEM (N.S., not significant).

Supplementary Figure 11. Quantitative analysis of the mRNA expression of proton-sensing GPCRs in cardiac macrophages.

Cardiac cells were isolated from WT mice on post-MI day 3, and cardiac macrophages (CD45.2^{+} Ly6G^{-} CD11b^{+} CD3^{-}) were sorted after eliminating dead cells and doublet cells. The levels of proton-sensing GPCRs (TDAG8, GPR4, OGR1 and G2A) were quantified using real-time RT-PCR. Three infarcted WT mice hearts were combined for this analysis.

Supplemental Table 1. Sequences of primers or Assay ID used for real time RT-PCR

mRNA	Sequences of primers (Sigma)
Il17a	Forward: 5′-GGTCAACCTCAAAGTCTTTAACTCC-3′
	Reverse: 5′-GGTCTTCATTGCGGTGGAGAG-3′
	Probe: 5′-CCAGAAGGCCCTCAGACTACCTCAACCG-3′
Ccl2	Forward: 5′-CGGCTGGAGCATCCACGT-3′
	Reverse: 5′-ATTGGGATCATCTTGCTGGTGAAT-3′
	Probe: 5′-TCAGCCAGATGCAGTTAACGCCCCAC-3′
Cxcl12	Forward: 5′-GTGACGGTAAACCAGTCAGCC-3′
	Reverse: 5′-GCACAGTTTGGAGTGTTGAGGA-3′
	Probe: 5′-CGGTTCTTCGAGAGCCACATCGCCAG-3′
Ccl20	Forward: 5′-CCTCTCGTACATACAGAC-3′
	Reverse: 5′-CGTGTGAAAGATGATAGC-3′
	Probe: 5′-CATCGGCCATCTGTCTTGTGAA-3′
Il23	Forward: 5′-TCAAGGACAACAGCCAGTTCT-3′
	Reverse: 5′-AAGATGTCAGAGTCAAGCAGGT-3′
	Probe: 5′-AGCCAGACCTTGGCGGATCCTTTGC-3′

Supplemental Table 1 (Continued)

**mRNA**	**Sequences of primers (Sigma)**
Tdag8	Forward: 5′-TGAGCTAGGGATTTACCTCTTCAG-3′
	Reverse: 5′-AGTCCAGTTGTCTTTATTCCAAGTG-3′
	Probe: 5′-CTGTCCCTGTCAGACCTGCTGTATGCG-3′
Gpr4	Forward: 5′-GCACCGCTCTTCCATGATGA-3′
	Reverse: 5′-CGCTCCATGGGGAACTTCTC-3′
	Probe: 5′-TCGTGATCGCTACAACCACACCTTCTGCT-3′
Ogr1	Forward: 5′-TCTGGGAGAGAAACTGTGAGTTTG-3′
	Reverse: 5′-TCCTCGGAGGCGGGCTAG-3′
	Probe: 5′-TCTATCACTTCTCCCTCCTCCTCACCAGCT-3′
G2a	Forward: 5′-GGTCCTGGTGGTGGTGTAC-3′
	Reverse: 5′-GCAGAACAGGTAGACGGCTAG-3′
	Probe: 5′-CCTACCAGCCAACTGCCTGACTGCCT-3′
GAPDH	Forward: 5′-CGTCCCGTAGACAAAATGGTGA-3′
	Reverse: 5′-CCACTTTGCCACTGCAAATGG-3′
	Probe: 5′-CCAATACGGCCAAATCCGTTCACACCGA-3′

Supplemental Table 1 (Continued)

**mRNA**	**Sequences of primers (Sigma) or Assay ID (Thermo Fisher)**
18S rRNA	Forward: 5′-GGGTCATAAGCTTGCGTTGATTAAG-3′
	Reverse: 5′-TCCGAGGGCCTCACTAAAC-3′
	Probe: 5′- TACACACCGCCCGTCGCTACTACCG-3′
Cxcl15 (*Il8*)	Forward: 5′- ACAGAAAGGAAGTGATAGCAGTCC-3′
	Reverse: 5′- GAGGTCCTCAGGTAGGAACCT-3′
	Probe: 5′- ATTGGGCCAACAGTAGCCTTCACCCATG-3′
Tnfα	Mm00443258_m1
Il1β	Mm00434228_m1
Il6	Mm00446190_m1
Ccl20	Mm01268754_m1
Col1a1	Mm00801666_g1
Tgfβ*1*	Mm01178820_m1
Ifnγ	Mm01168134_m1
Tdag8	Mm02619732_s1
18S rRNA	Hs03003631_g1
Csf-2	Mm01290062_m1

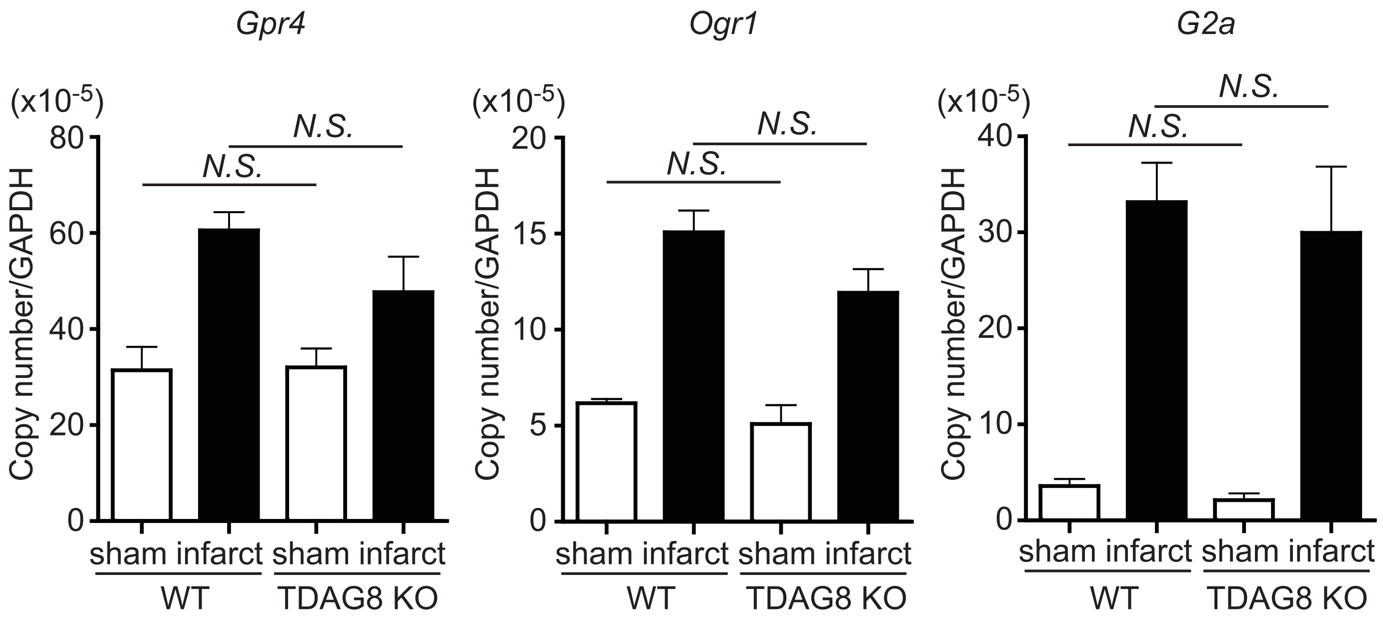

Supplementary Figure 1. Comparison of Gpr4, Ogr1 and G2a expression levels in the hearts of WT and TDAG8 KO mice on post-MI day 3.
Quantitative analysis of the expression of proton-sensing GPCR mRNAs in WT mice hearts (sham, n = 3; MI, n = 7) and TDAG8 KO mice hearts (sham, n = 3; MI, n = 5) on post-MI day 3. mRNA expression was normalized to GAPDH. Comparisons were assessed using unpaired Student's *t*-tests. Error bars represent the mean ± SEM (N.S., not significant).

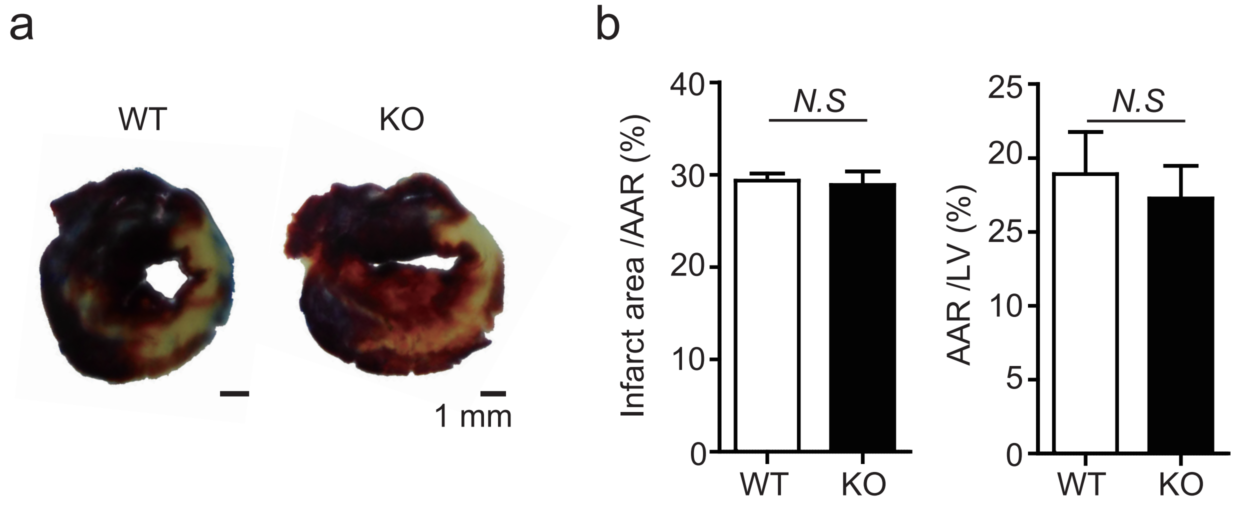

Supplementary Figure 2. The area at risk and infarct size were comparable between WT and TDAG8 KO mice during the initial phase after MI.

(a) Representative heart sections of Evans blue/TTC staining 3 h after MI. The non-ischemic area is indicated in blue, the area at risk (AAR) in red and the infarct area in white. (b) The infarct size and AAR were quantified as a percentage of the AAR and the left ventricular area (LV) of WT (n = 5) and TDAG8 KO (n = 4) mice. Error bars represent the mean ± SEM. Comparisons among groups were assessed using unpaired Student's *t*-test (N.S., not significant).

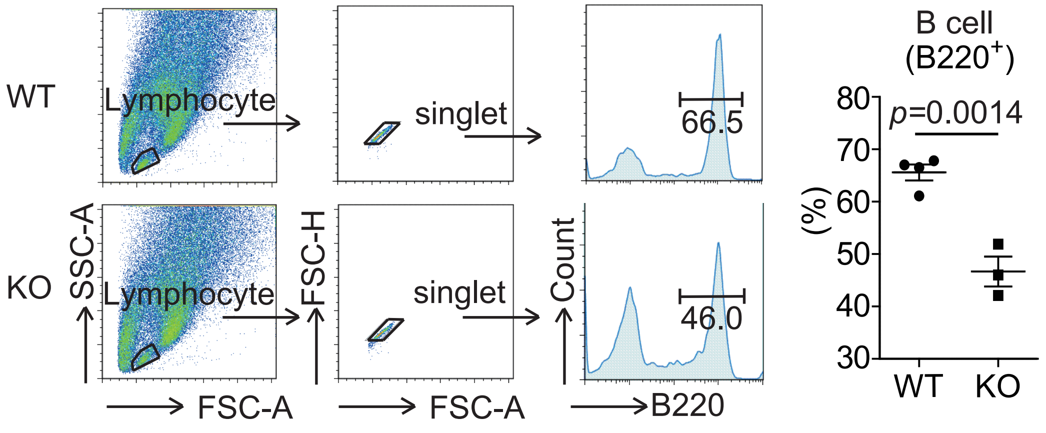

Supplementary Figure 3. Comparison of infiltrated B cells in the infarcted hearts of WT and TDAG8 KO mice.

Comparison of infiltrated B cells (B220+) on post-MI day 3 between WT mice (n = 4) and TDAG8 KO mice (n = 3) was performed using flow cytometry. Representative FACS profiles are shown. The comparison was assessed using unpaired Student's t-tests. Error bars represent the mean ± SEM.

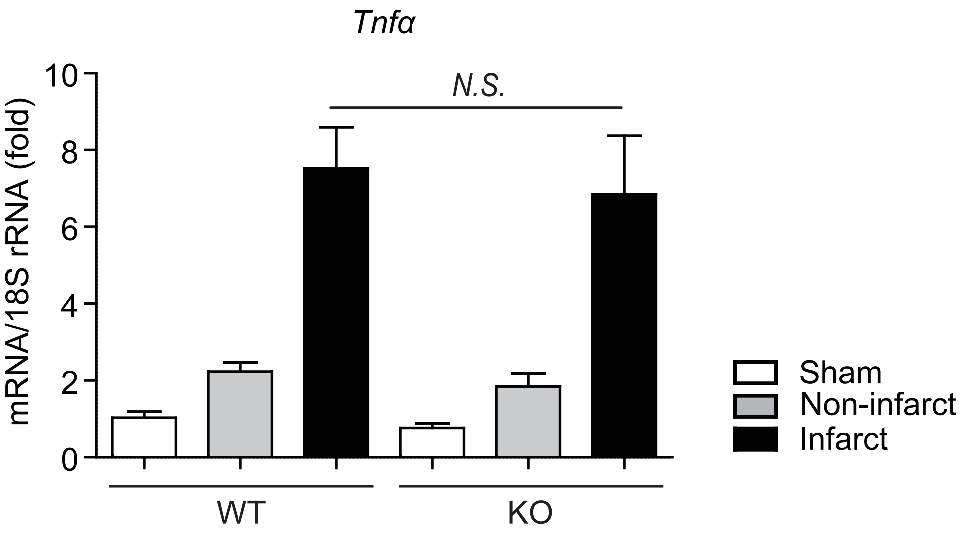

Supplementary Figure 4. No significant differences in *Tnfα* mRNA were found on post-MI day 3.
Tnfα mRNA expression in infarcted and non-infarcted areas of WT mice (sham n = 3, MI n = 7) and TDAG8 KO mice (sham n = 3, MI n = 5) 3 days after MI was measured using real-time RT-PCR. The comparison was assessed using an unpaired Student's *t*-test.

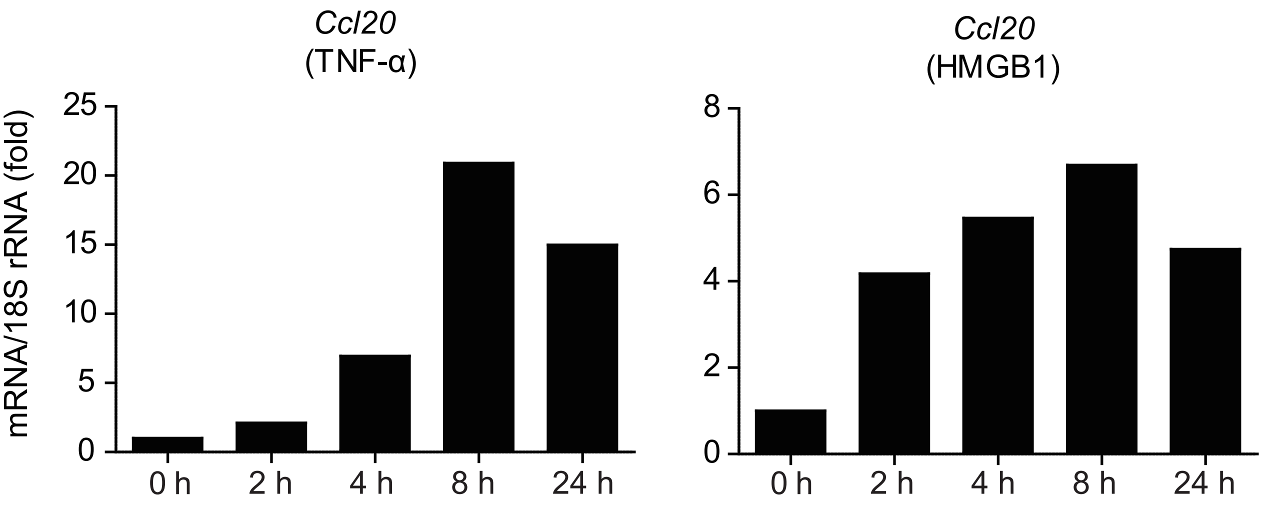

Supplementary Figure 5. Time course of *Ccl20* mRNA expression in cardiac macrophages stimulated with TNF-*α* and HMGB1.
Cardiac macrophages were isolated from WT mice on post-MI day 3 and stimulated with 10-ng/ml TNF-*α* (left) or 10-μg/ml HMGB1 (right). Three heart samples were combined as one sample and used for real-time RT-PCR analysis.

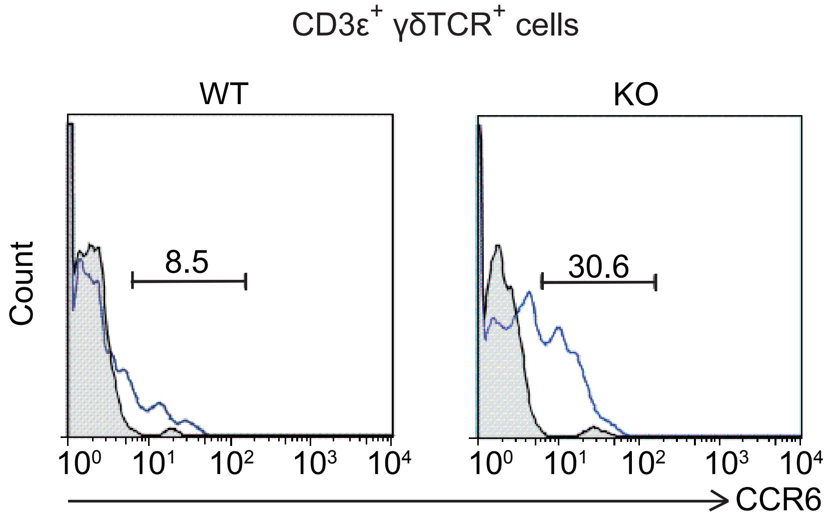

Supplementary Figure 6. Comparison of infiltrated CCR6+ γδT cells in the infarcted hearts of WT and TDAG8 KO mice on post-MI day 5.
Cardiac γδT cells were isolated from WT and TDAG8 KO mice 5 days after MI; stained with antibodies against CD3ε, γδTCR and CCR6; and were analysed by flow cytometry. Grey histograms indicate the isotype control.

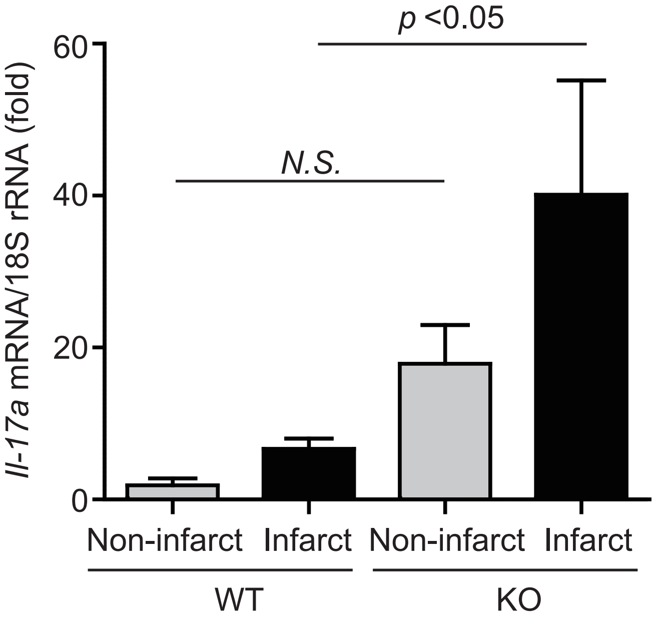

Supplementary Figure 7. Upregulation of *Il-17a* mRNA in TDAG8 KO mice 7 days after MI. *IL-17a* mRNA expression in the non-infarct and infarcted areas of WT mice (n = 7) or TDAG8 KO mice (n = 5) 7 days after MI was measured using real-time RT-PCR. Comparisons were assessed with a one-way ANOVA followed by Tukey's test. Error bars represent the mean ± SEM (N.S., not significant).

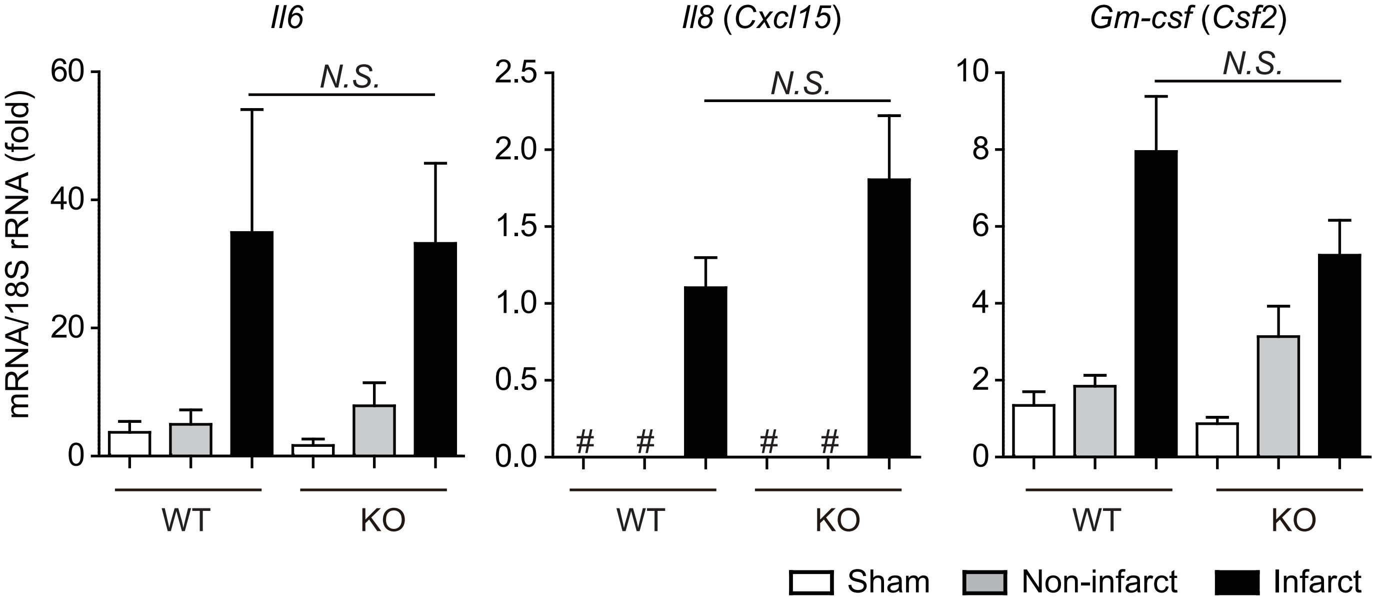

Supplementary Figure 8. Comparison of IL-17A-related inflammatory genes of WT and TDAG8 KO mice.

Expression levels of inflammatory gene mRNAs in the infarcted and non-infarcted areas of WT mice (sham, n = 6; MI, n = 10) and TDAG8 KO mice (sham, n = 5; MI, n = 8) on post-MI day 3. # indicates that Il8 mRNA was not detected. Comparisons were assessed using unpaired Student's *t*-tests. Error bars represent the mean ± SEM (N.S., not significant).

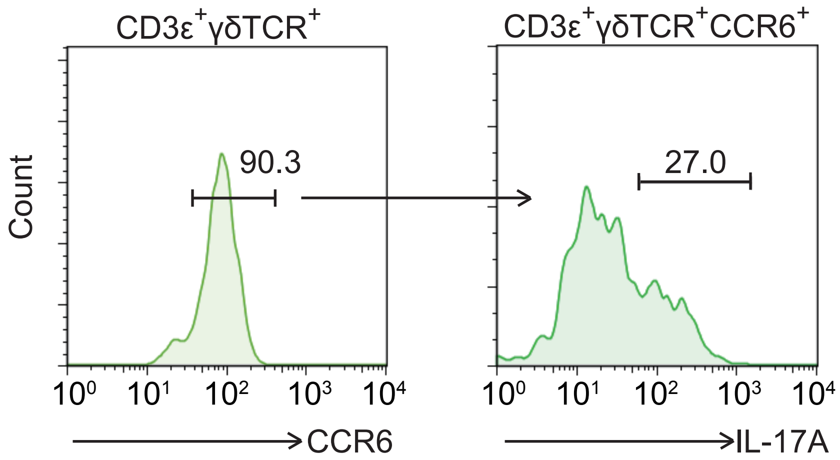

Supplementary Figure 9. A portion of CCR6$^+$ γδT cells secrete IL-17A.
Cardiac cells were isolated from TDAG8 KO mice on post-MI day 5 and stimulated with PMA and ionomycin in the presence of Brefeldin A. The samples were stained with antibodies against CD3ε, γδTCR, CCR6 and IL-17A. After gating visible and singlet cells, CD3ε$^+$ γδTCR$^+$ CCR6$^+$ cells were further gated, and IL-17A expression was determined using flow cytometry. Mixed samples (n = 3) were used for the a.nalysis.

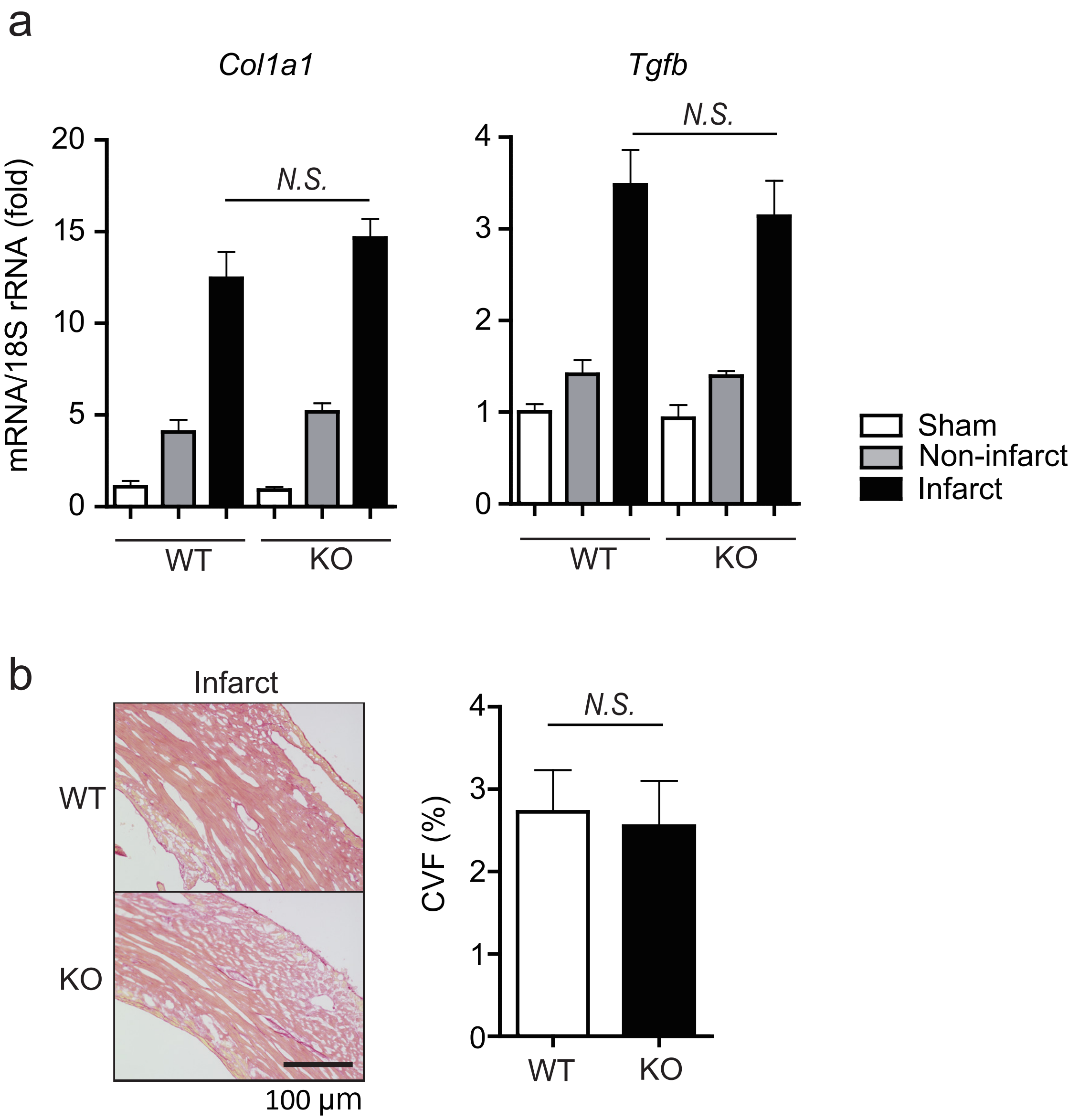

Supplementary Figure 10. Comparison of fibrosis between WT and TDAG8 KO mice on the post-MI day 3.

(a) Expression levels of fibrosis-related gene mRNAs in the infarcted and non-infarcted area of WT mice (sham n = 3, MI n = 7) and TDAG8 KO mice (sham n = 3, MI n = 5) on post-MI day 3. (b) Quantification of the fibrotic area using Picrosirius Red staining in the infarcted areas on post-MI day 3 in WT (n = 3) and TDAG8 KO (n = 3) mice. Comparisons were assessed using unpaired Student's *t*-tests. Error bars represent the mean ± SEM (N.S., not significant).

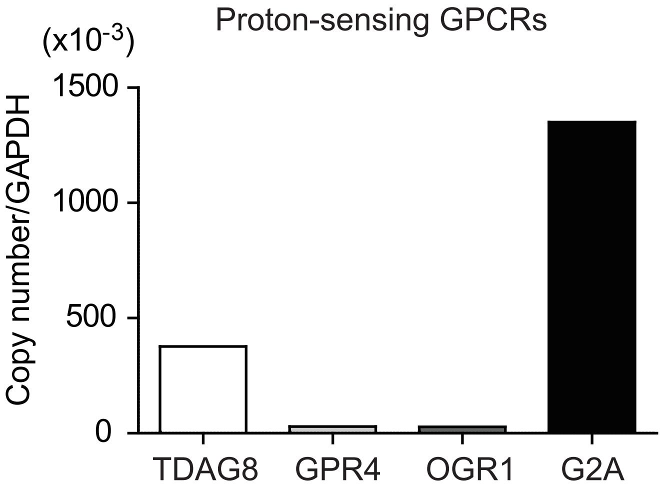

Supplementary Figure 11. Quantitative analysis of the mRNA expression of proton-sensing GPCRs in cardiac macrophages.

Cardiac cells were isolated from WT mice on post-MI day 3, and cardiac macrophages ($CD45.2^{+}$ $Ly6G^{-}$ $CD11b^{+}$ $CD3^{-}$) were sorted after eliminating dead cells and doublet cells. The levels of proton-sensing GPCRs (TDAG8, GPR4, OGR1 and G2A) were quantified using real-time RT-PCR. Three infarcted WT mice hearts were combined for this analysis.
